# Diagnostic NGS for Severe Neuromuscular Disorders

**DOI:** 10.15844/pedneurbriefs-29-11-1

**Published:** 2015-11

**Authors:** Radhika Dhamija, Chelsea Chambers

**Affiliations:** 1 Division of Pediatric Neurology, Department of Neurology, University of Virginia, Charlottesville, VA

**Keywords:** Next-Generation-Sequencing, Novel Genes, Neuromuscular Disorders

## Abstract

Investigators from the University of Western Australia report the diagnostic yield of performing next generation sequencing (NGS; whole exome and targeted capture of 277 neuromuscular genes) in a heterogenous cohort of patients with neuromuscular disorders (NMD) presenting at or before birth.

Investigators from the University of Western Australia report the diagnostic yield of performing next generation sequencing (NGS; whole exome and targeted capture of 277 neuromuscular genes) in a heterogenous cohort of patients with neuromuscular disorders (NMD) presenting at or before birth. Forty-five patients from 38 unrelated families with fetal akinesia (9 families), arthrogryposis (13 families) and severe congenital myopathies (16 families) underwent whole exome sequencing (23), targeted sequencing (7) and both (8). Ten of these families were consanguineous. A conclusive genetic diagnosis was achieved in 18/38 families (47%). Autosomal recessive was the most common mode of inheritance (15), however dominant (1), de novo (1) and X linked (1) were also identified. Mutations were found in eight previously known neuromuscular disease genes (CHRND, CHNRG, ECEL1, GBE1, MTM1, MYH3, NEB and RYR1) and four novel neuromuscular disease genes (GPR126, KLHL40, KLHL41 and SPEG).

This study highlights the widening spectrum of phenotypes associated with mutations in known neuromuscular genes. For example, null mutations in the *RYR1* are associated with the arthrogryposis and fetal akinesia phenotype while missense mutations in *RYR1* are associated with central core myopathy phenotype (allelic heterogeneity). The study also led to the identification of novel neuromuscular disease genes (*KLHL40*, *KLHL41*, and *LMOD3)* involved in sarcomere assembly and muscle dysfunction. [1]

COMMENTARY. Fetal akinesia deformation sequence (intrauterine growth retardation, contractures, pulmonary hypoplasia and polyhydramnios), arthrogryposis (non-progressive congenital joint contractures in >1 area of the body), and severe congenital myopathies comprise a very heterogeneous group, both phenotypically and genetically, that present at or before birth [1]. The authors used NGS technology to study this diverse group. NGS has enabled sequencing of large numbers of genes in a single reaction and thus has enabled novel disease gene discovery. This technology has been used to sequence large panels of genes, whole exome, or whole genome [2]. Prior to NGS-based panel testing, patients would undergo a battery of invasive and expensive tests, often without obtaining a diagnosis [3].

This study used a combination of whole exome sequencing and targeted exome sequencing of known neuromuscular genes. Functional studies were done in cases of novel genes when feasible [1]. A previous study using comprehensive panel based testing, in patients with a variety of NMD's, reported a yield of 46% (3 fold greater than single gene testing) [3]. For genetically and phenotypically heterogeneous disorders like NMD's, targeted panel based sequencing should be the first step. If negative, it should be followed by exome sequencing to increase the diagnostic yield. This principle has helped find a genetic basis in a variety of undiagnosed neurogenetic disorders in a cost and time effective manner [4]. This approach also helps overcome some unique diagnostic challenges in NMD's such as: genetic heterogeneity (large number of causative genes), phenotypic heterogeneity (multiple genes with overlapping phenotype or a single gene with multiple phenotypes) and allelic heterogeneity (variety of mutations in each gene) [1, 3].
